# Prognostic value of immune-related genes and comparative analysis of immune cell infiltration in lung adenocarcinoma: sex differences

**DOI:** 10.1186/s13293-021-00406-y

**Published:** 2021-12-04

**Authors:** Tao Fan, Chunxiang Li, Jie He

**Affiliations:** 1 Department of Oncology, Renmin Hospital of Wuhan University, 238th Jiefang Road, Wuhan, 430060 China; 2 Department of Thoracic Surgery, National Cancer Center/National Clinical Research Center for Cancer/Cancer Hospital, Chinese Academy of Medical Sciences and Peking Union Medical College, Beijing, 100021 China

**Keywords:** Lung adenocarcinoma, Sex differences, Tumor microenvironment, ESTIMATE algorithm, Tumor-infiltrating immune cells

## Abstract

**Background:**

Lung adenocarcinoma (LUAD) is one of the most important subtypes of lung cancer. Compared with male LUAD patients, female patients have a higher incidence, but better long-term survival rate, with unknown reasons. In this study, we aimed to explore the effect of sex differences on immune cell infiltration in lung tumor microenvironment (TME), and tried to clarify the reasons for the different clinical characteristics of male and female LUAD patients, by conducting a comparative analysis of the TME.

**Methods:**

Using ESTIMATE algorithm, we calculated immune and stromal scores of tumor samples downloaded from TCGA database according to immune or stromal components in TME. GO and KEGG enrichment analysis were conducted to reveal biological processes of these intersecting genes of high- and low-score groups. Cox regression analysis and protein–protein interaction (PPI) network analysis were performed to screen immune-related prognostic genes in female (CCR2, LCP2, and PTPRC) and male (BTK and CCR2) patients. Kaplan–Meier survival analysis was used to evaluate prognostic value of these identified genes. Mann–Whitney test was used to compare various indicators of male patients and female patients. The main results were subsequently validated in 420 cases from GSE72094.

**Results:**

304 and 368 intersecting genes were identified in female and male patients, respectively. The immune score ranged from −943.17 to 3229.35 among female patients and from −541.75 to 3441.78 among male patients. The stromal score ranged from −1790.23 to 2097.27 among female patients and from −1786.94 to 1722.70 among male patients. The immune and stromal scores of women were higher than those of men (*p* < 0.05). CCR2, LCP2 and PTPRC were identified as the most important immune-related prognostic genes in female LUAD patients. BTK and CCR2 were identified as the most important immune-related prognostic genes in male LUAD patients. Female patients had a higher proportion of memory B cells than that of male patients, while the percentage of T cells CD4 naïve and resting NK cells was lower in female patients (*p* < 0.05).

**Conclusions:**

This study comprehensively compared the differences in tumor immune microenvironment between male and female LUAD patients, and identified prognosis-related genes for patients of different sexes.

**Supplementary Information:**

The online version contains supplementary material available at 10.1186/s13293-021-00406-y.

## Abstract (articlehighlights)

1. We presented a detailed and comprehensive analysis of immune cell infiltration in tumor microenvironment (TME) of female and male patients with lung adenocarcinoma (LUAD).
2. We compared the differences in the tumor immune microenvironment between female and male LUAD patients.
3. The density of memory B cells in TME of female patients with LUAD was significantly higher than that of male patients, which may explain that female patients with LUAD have a better prognosis than that of male patients.
4. We identified immune-related genes that predicted the prognosis of female and male LUAD patients, respectively, and their predictive value were verified by another independent cohort.

**Supplementary Information:**

The online version contains supplementary material available at 10.1186/s13293-021-00406-y.

## Background

As the most commonly diagnosed cancer, lung cancer is the leading cause of cancer death with a 5-year overall survival rate of 7.5% in males and 8.5% in females [1, 2]. Among all histologic subtypes of lung cancer, lung adenocarcinoma (LUAD) is the most prevalent type, accounting for more than 40% of all lung cancer [3]. Sex differences are involved in tumorigenicity and play an important role in tumor treatment [4–6]. There are sex differences in the occurrence and progression of LUAD, but so far the reason is unclear. This study was the first to compare the differences in tumor-infiltrating immune cells (TIICs) in TME between female and male LUAD patients, to explore the influence of sex differences on LUAD.

Recent studies have shown that components in TME, including immune cells, endothelial cells, mesenchymal cells, inflammatory mediators and extracellular matrix, were closely related to the occurrence, progression and prognosis of cancer [7–12]. As the two main components, TIICs and stromal cells were regarded as valuable factors affecting the effect of tumor immunotherapy [13–17]. It was reported that immune cells, such as T and B cells, macrophages, dendritic cells, monocytes, etc., interacted with tumor cells, were correlated to tumor cell proliferation, metastasis and prognosis [18–20]. Regulated by Tregs and M2 macrophages, tumor-infiltrating lymphocytes (TILs) were involved in the occurrence and progression of various cancers [21–25]. Donnem and his colleagues found that the density of CD8 (+) TIL was an independent prognostic factor in non-small cell lung cancer (NSCLC) [26]. In the study of colorectal cancer, it was found that the level of DNA microsatellite instability was related to the density of TIL. High-level microsatellite instability was characterized by the presence of TILs and favourable prognosis [27]. Furthermore, TME also had an impact on tumor-related gene expression and clinical prognosis [28–31]. These findings indicate that TME is closely related to tumor progression, which will provide a potential cure for cancer.

Estimation of Stromal and Immune cells in MAlignant Tumors using Expression data (ESTIMATE) was created by Yoshihara and his colleagues, which has been used to score the TME of various types of cancer [32]. For example, the ESTIMATE algorithm was reported to be applied to evaluate immune and stromal scores to predict the proportion of immune cells, stromal cells and other non-tumor cells in TME of lung cancer [33], breast cancer [34], uveal melanoma [35], gastric cancer [36], or colorectal cancer [37].

As a clinical variable, sex difference plays a significant role in tumor progression, which should be considered as an important factor in pathogenesis of cancer. Women tend to have lower mortality rates of lung cancer than that of men. In our study, we used gene expression profile from The Cancer Genome Atlas (TCGA) database to comparatively analyze the difference of TIICs and tumor-related genes between female and male patients with LUAD based on ESTIMATE algorithm, to explore the reasons that male patients had a worse prognosis than that of female patients.

## Methods

### Data mining from the public database

Gene expression data of female (*n* = 304) and male (*n* = 247) patients with LUAD were downloaded from the data portal of TCGA (https://tcga-data.nci.nih.gov/tcga/), which included complete clinical information (age, TNM stage, survival and outcome). Estimate score, immune score and stromal score were calculated by ESTIMATE algorithm. GSE72094 cohort contained gene expression data of 232 female and 188 male LUAD patients, which was downloaded from Gene Expression Omnibus (GEO) (http://www.ncbi.nlm.nih.gov/geo) to serve as validation set.

### Identification of differentially expressed genes

Differentially expressed genes (DEGs) in high- and low-score groups were screened using the “limma” package [38]. A |logFC|> 1 and an adjusted *p* < 0.05 were set as the cutoff criteria. In addition, “heatmap” package was used to construct heat maps.

### Survival analysis

Patients were divided into high- and low-score (or gene expression) groups by the medium value. Differences in overall survival between high- and low-score (or gene expression) groups were analyzed using “survival” and “survminer” packages.

### Functional analysis and cox analysis of DEGs

Gene Ontology (GO) and Kyoto Encyclopedia of Genes and Genomes (KEGG) enrichment analysis for DEGs between high- and low-score groups in LUAD patients with different sexes was conducted using “clusterProfiler”, “org.Hs.eg.db”, “enrichplot” and “ggplot2” packages. The *p* < 0.05 and *q* < 0.05 were considered to be statistically significant. At the same time, we performed univariate Cox regression analysis on DEGs to screen prognostic factors that regulated TME and tumor immunity.

### PPI analysis

The protein–protein interaction (PPI) networks were analyzed by an online tool named Search Tool for the Retrieval of Interacting Genes (STRING) [39]. Confidence Score ≥ 0.9 was set as the cutoff value. We analyzed the connectivity degree of each DEGs using STRING database and reconstructed the networks via Cytoscape software.

### TME analysis

CIBERSORT is a deconvolution algorithm that has been widely used to calculate the numbers of each type of TIICs and analyze the correlation between gene expression and TIICs in TME [40–43]. The “corrplot” package was used to conduct correlation-based heatmaps.

### Statistical analysis

Kaplan–Meier plots were used to analyze and visualize the associations of estimate, immune, and stromal scores and DEGs with prognosis. The correlations of estimate, immune, and stromal scores with age and TNM stage were estimated via Wilcoxon test or Mann–Whitney U tests. The estimate, immune, and stromal scores of female LUAD patients were compared with that of male LUAD patients using GraphPad Prism 8. Functional analysis, Cox analysis, survival, and TME analysis were conducted using R version 3.6.2. A two-sided *p* < 0.05 was considered to be statistically significant.

## Results

### Comparison of TME scores and gene expression profiles in patients with LUAD of different sexes

**Fig. 1:**
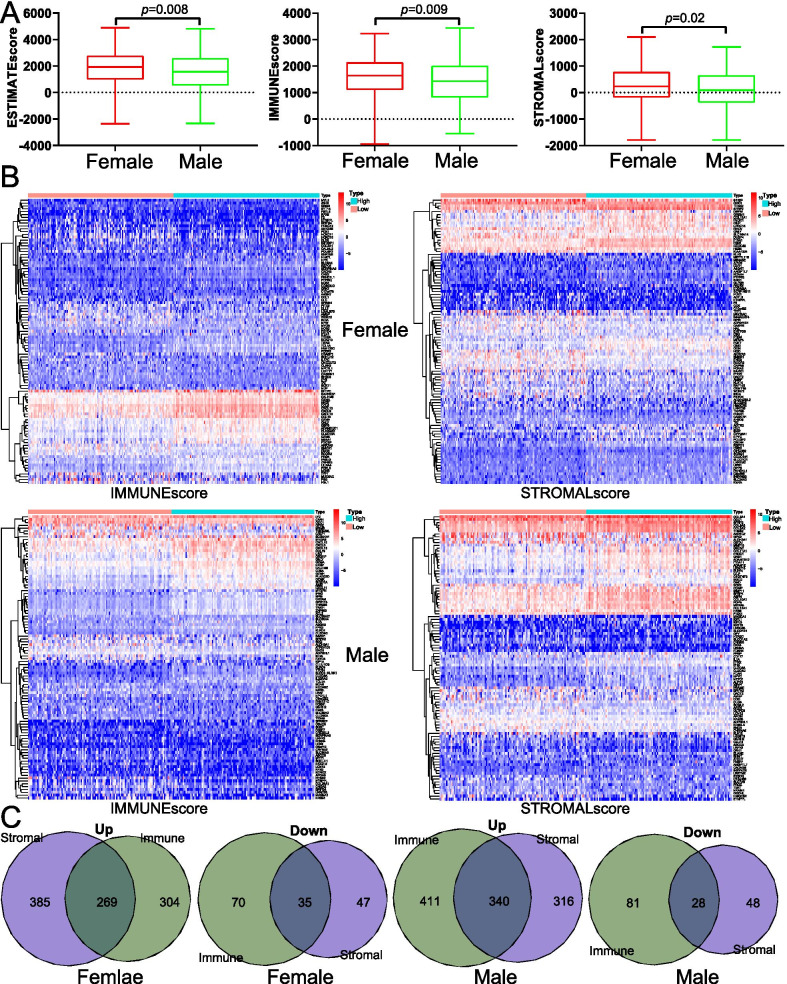
Comparison of tumor microenvironment scores and gene expression profiles in patients with lung adenocarcinoma (LUAD) of different sexes. **A** Box-plot comparing the levels of estimate score, immune score, and stromal score of female patients with male patients. **B** Heatmaps of differentially expressed genes (GEGs) between high- and low-estimate, immune and stromal scores in LUAD. Red represented high expression, and blue represented low expression. **C** Venn diagram analysis of DEGs in female and male patients

Gene expression data and corresponding clinical information were downloaded from TCGA database, including 304 cases of female LUAD patients and 247 cases of male LUAD patients. The estimate score ranged from −2358.46 to 4889.83 among female patients and from −2328.69 to 4818.63 among male patients. The immune score ranged from −943.17 to 3229.35 among female patients and from −541.75 to 3441.78 among male patients. The stromal score ranged from −1790.23 to 2097.27 among female patients and from −1786.94 to 1722.70 among male patients. The estimate, immune and stromal scores of female patients were higher than those of male patients (*p* < 0.05) (Fig. 1A). The samples were divided into high- and low-score groups by the medium value. Up-regulated genes and down-regulated genes were showed in Fig. 1B. By interaction of DEGs in immune and stromal groups, 269 up-regulated genes and 35 down-regulated genes were screened in female patients, and 340 up-regulated genes and 28 down-regulated genes were screened in male patients (Fig. 1C).

### Associations of these scores with age, TNM stage and prognosis in LUAD patients of different sexes

**Fig. 2:**
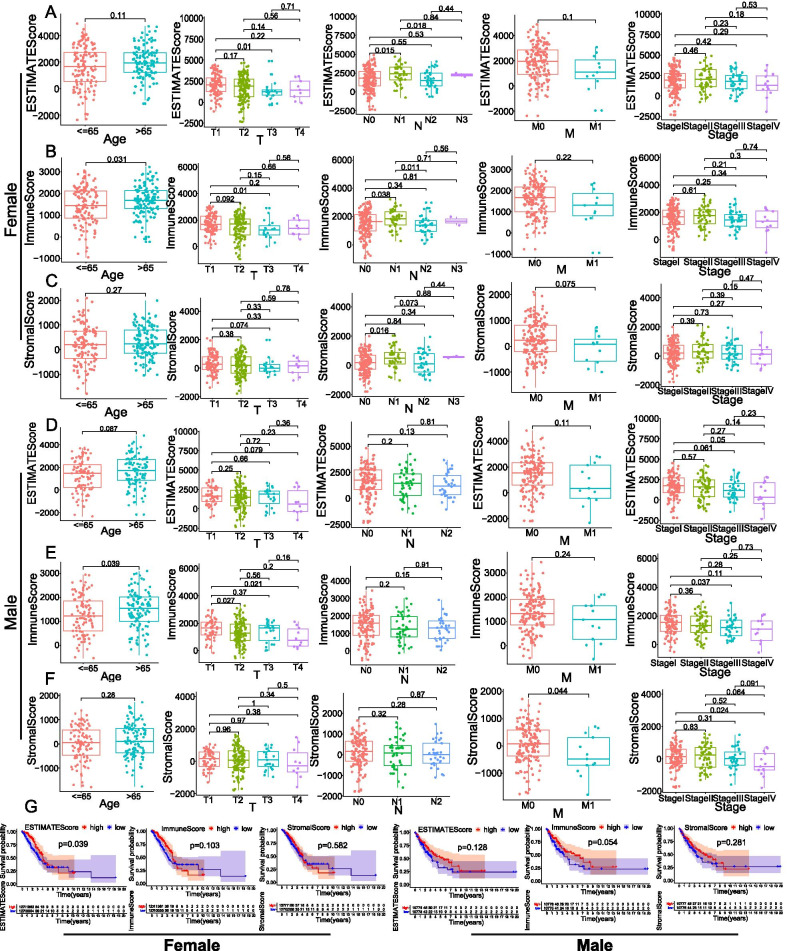
Associations of tumor microenvironment scores with age, TNM stage and prognosis in LUAD patients of different sexes. All the female and male LUAD cases were divided into high or low score groups based on a median score. Box-plots depicting the relationship of estimate score (**A**), immune score (**B**), and stromal score (**C**) with age and TNM stage in female patients. Box-plots depicting the relationship of estimate score (**D**), immune score (**E**), and stromal score (**F**) with age and TNM stage in male patients. **G** Kaplan–Meier plots of different estimate score, immune score, and stromal score in female and male patients. A p < 0.05 was considered to be statistically significant

To explore the relationship between estimate, immune or stromal scores and age, TNM stage or survival, the samples were divided into high-score and low-score groups. The analysis indicated that, for female patients with LUAD, T1 stage had higher estimate score than that of T3 stage (*p* = 0.01), and N1 stage had higher estimate score than that of N0 stage (*p* = 0.015) and N2 stage (*p* = 0.018) (Fig. 2A). However, in male patients with LUAD, estimate score had no obvious relationship with the T or N stage (Fig. 2D). Whether it was a male or female patient with LUAD, high immune score was related to older age (Fig. 2B, E). Immune score of T1 stage was higher than that of T3 stage in female patients (Fig. 2B), but in male patients, immune score of T1 stage was higher than that of T2 and T4 stage (Fig. 2E). In addition, N1 stage had higher immune score than that of N0 stage (*p* = 0.038) and N2 stage (*p* = 0.011) (Fig. 2B) for female patients, and clinical stage I had higher immune score than that of stage III for male patients (*p* = 0.037) (Fig. 2E). For female patients with LUAD, the stromal score of N1 stage was higher than that of N0 (*p* = 0.016) (Fig. 2C), and stromal score of M0 stage or clinical stage I were higher than that of M1 stage (*p* = 0.044) or clinical stage IV (*p* = 0.024) (Fig. 2F). To study the correlations of overall survival with estimate, immune or stromal scores, we performed Kaplan–Meier survival analysis (Fig. 2G). The results indicated that high estimate score had better overall survival for female patients with LUAD (*p* = 0.039), but they had no significant relationship with prognosis of male patients (*p* = 0.128). The overall survival was not associated with immune or stromal score whether it was a male patient or a female patient (*p* > 0.05).

### GO and KEGG analysis of immune-related DEGs

**Fig. 3:**
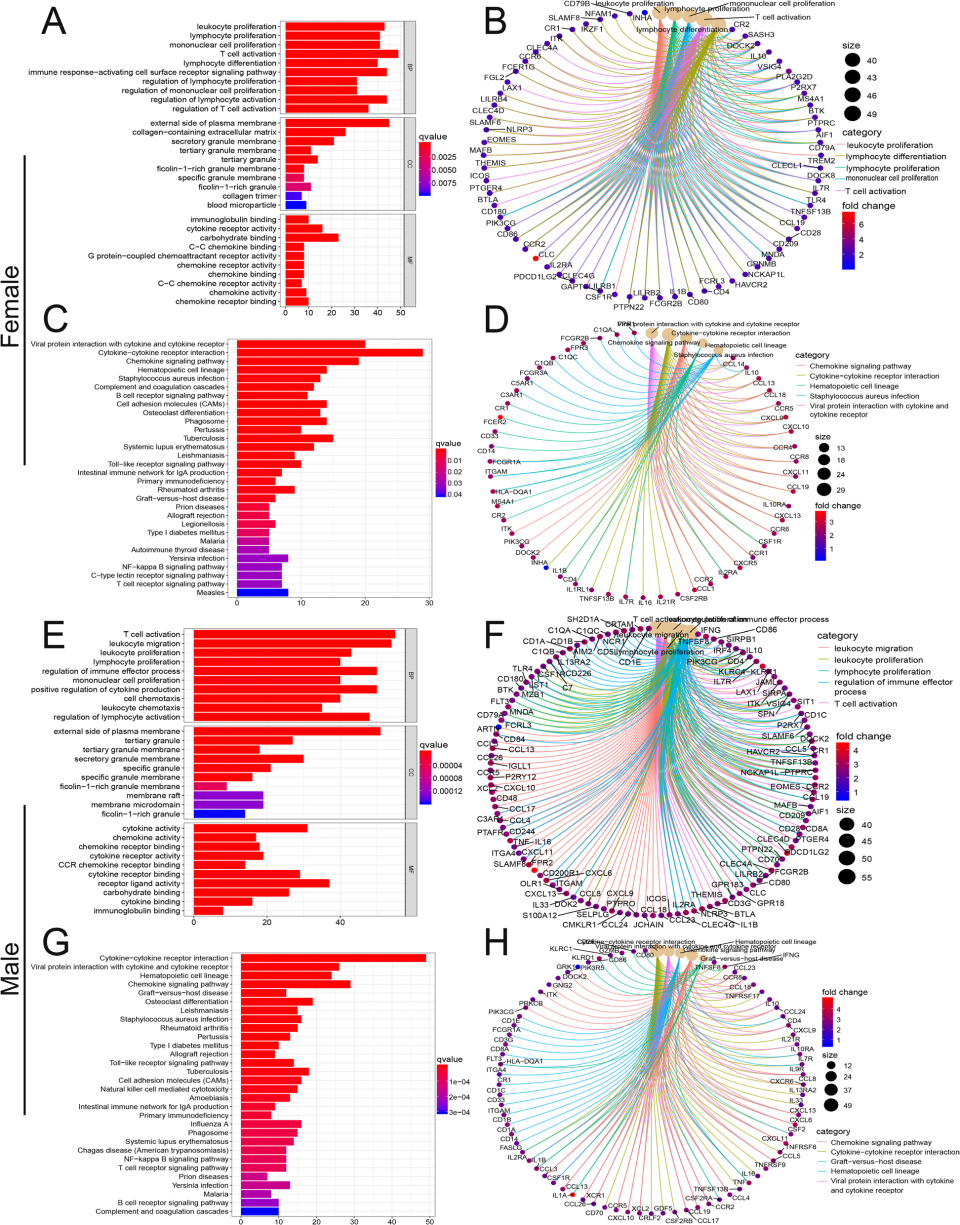
GO and KEGG analysis of immune-related DEGs based on estimate score, immune score, and stromal score in female and male patients. **A**, **C** GO analysis of DEGs in female patients. **B**, **D** Circular plot demonstrating that the functional interactions between the BP, CC or MF pathways and genes extracted from GO in female patients. **E**, **G** GO analysis of DEGs in male patients. **F**, **H** Circular plot demonstrating the functional interactions between the BP, CC or MF pathways and genes extracted from GO in male patients. GO, Gene Ontology; KEGG, Kyoto Encyclopedia of Genes and Genomes; BP, biological process; CC, cellular component; MF, molecular function

To further explore the functions of immune-related DEGs, we performed GO and KEGG analysis on these genes. The top 5 enriched biological process (BP) pathways in female patients with LUAD based on GO analysis were leukocyte proliferation, lymphocyte proliferation, mononuclear cell proliferation, T-cell activation, and lymphocyte differentiation (Fig. 3A), and the top 5 enriched BP pathways in male patients with LUAD based on GO analysis were T-cell activation, leukocyte migration, leukocyte proliferation, lymphocyte proliferation, and regulation of immune effector process (Fig. 3E). Circular plot demonstrated the functional interactions between the BP, cellular component (CC) or molecular function (MF) pathways and genes extracted from GO (Fig. 3B, F). Moreover, the top 5 enriched BP pathways in female patients with LUAD based on KEGG were viral protein interaction with cytokine and cytokine receptor, cytokine–cytokine receptor interaction, chemokine signaling pathway, hematopoietic cell lineage, and staphylococcus aureus infection (Fig. 3C), and top 5 enriched BP pathways in male patients with LUAD based on KEGG were cytokine–cytokine receptor interaction, viral protein interaction with cytokine and cytokine receptor, hematopoietic cell lineage, chemokine signaling pathway, and graft-versus-host disease (Fig. 3G). Circular plot demonstrated the functional interactions between the BP, CC or MF pathways and genes extracted from KEGG (Fig. 3D, H).

### PPI network and Cox regression analysis of immune-related DEGs

**Fig. 4:**
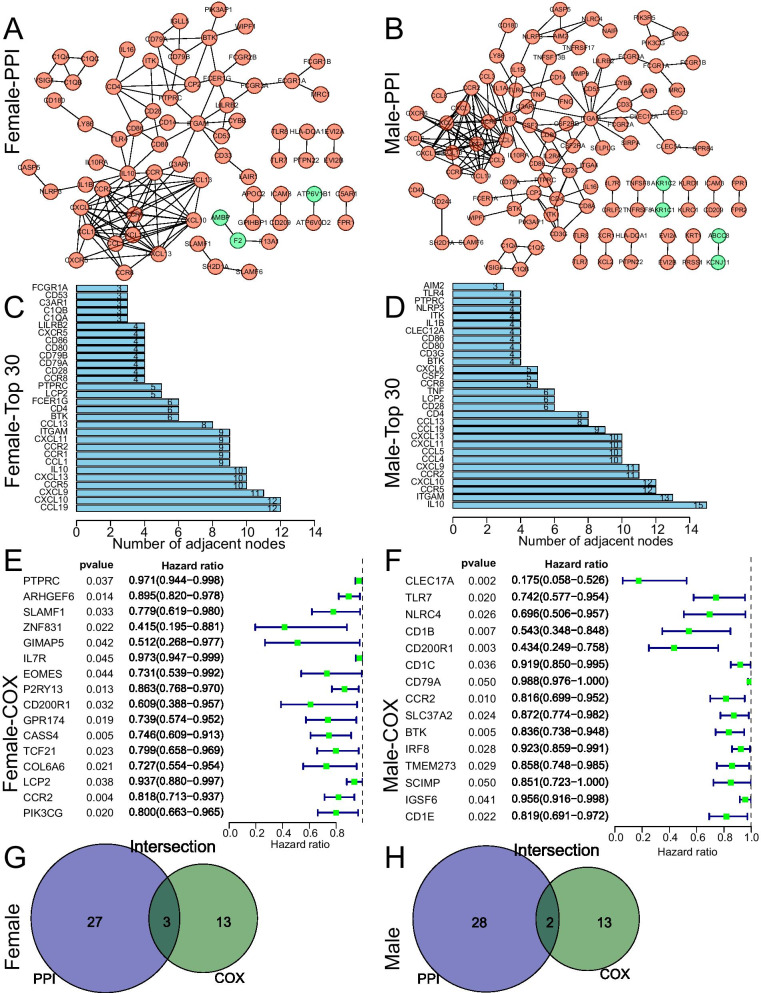
PPI network and Cox regression analysis of immune-related DEGs. **A**, **B** PPI networks of the immune-related DEGs plotted by Cytoscape in female and male patients with LUAD. **C**, **D** Top 30 genes with most number of adjacent nodes in female and male patients with LUAD. **E**, **F** Univariate Cox analysis of the immune-related DEGs in female and male patients with LUAD, and genes with p < 0.05 displayed in forest plots. **G**, **H** Two-way Venn diagram comparing the key genes in female and male groups. CCR2, LCP2, and PTPRC were selected as prognostic factors of female patients with LUAD. BTK and CCR2 were screened as prognostic factors of male patients with LUAD. A p < 0.05 was considered to be statistically significant

The STRING tool was used to plot PPI networks of immune-related DEGs, which were regenerated by Cytoscape (Fig. 4A, B). The top 30 genes with most number of adjacent nodes in female and male patients with LUAD were showed in Fig. 4C and D. We performed univariate Cox regression analysis to evaluate the prognostic value of immune-related DEGs in female (Fig. 4E) and male (Fig. 4F) patients (genes with *p* < 0.05 displayed in forest plot). Two-way Venn diagram identified the key genes in female (Fig. 4G) and male (Fig. 4H) cohorts. CCR2, LCP2, and PTPRC were selected as key prognostic factors of female patients. BTK and CCR2 were selected as key prognostic factors of male patients.

### The expression level of these key TIICs-related genes and their prognostic value in LUAD patients

**Fig. 5:**
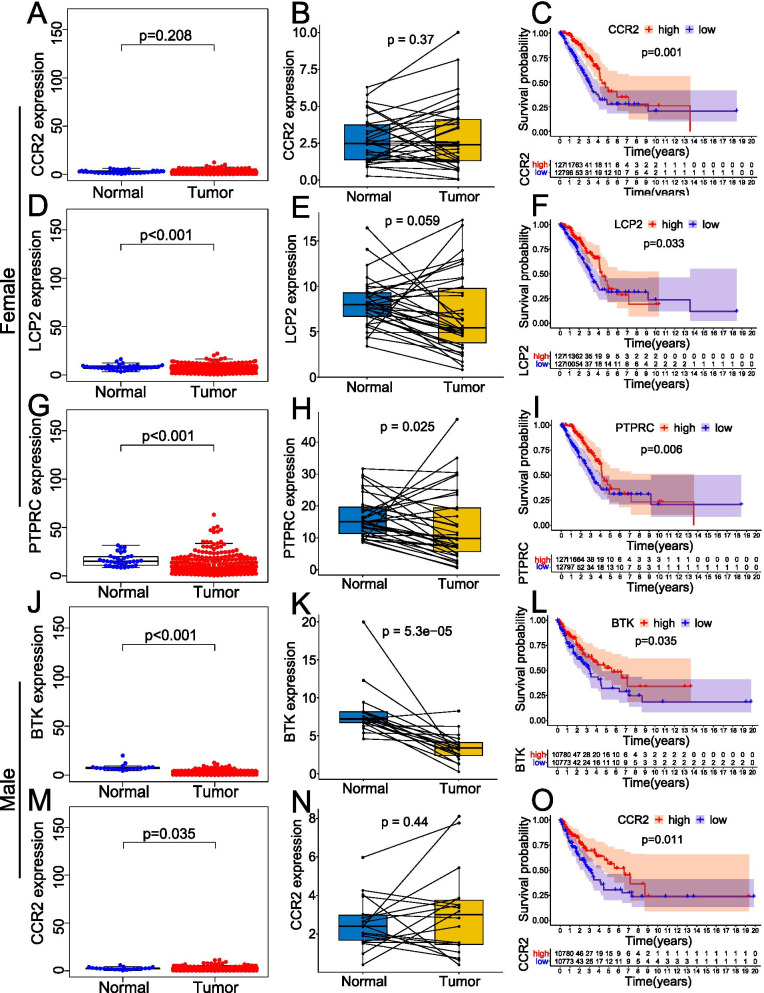
Expression level of these key TIICs-related genes and their prognostic value in LUAD patients. Box-plots comparing the expression differences of CCR2 (**A**), LCP2 (**D**), and PTPRC (**G**) in normal tissues and LUAD tissues of female patients. Box-plots comparing the expression differences of CCR2 (**B**), LCP2 (**E**), and PTPRC (**H**) in paired tumor and adjacent normal tissues of female patients. Kaplan–Meier plots of different CCR2 (**C**), LCP2 (**F**), and PTPRC (**I**) expression levels in female patients. Box-plots comparing the expression differences of BTK (**J**) and CCR2 (**M**) in normal tissues and LUAD tissues of male patients. Box-plots comparing the expression differences of BTK **(K)** and CCR2 (**N**) in paired tumor and adjacent normal tissues of male patients. Kaplan–Meier plots of different BTK (**L**) and CCR2 (**O**) expression levels in male patients. A p < 0.05 was considered to be statistically significant

We further revealed the expression level of these key TIICs-related genes and their prognostic value in LUAD patients. According to the results, the expression level of CCR2 in tumor tissues of female LUAD patients was not significantly different from that in normal tissues (*p* > 0.05) (Fig. 5A and B), but female LUAD patients with high level of CCR2 exhibited a better overall survival (*p* = 0.001) (Fig. 5C). The expression level of LCP2 in tumor tissues of female LUAD patients was lower than that in normal tissues (*p* < 0.05) (Fig. 5D), yet there was no difference in the expression of LCP2 between paired tumors and adjacent normal tissues (*p* > 0.05) (Fig. 5E). Interestingly, female LUAD patients with a high level of LCP2 exhibited a better overall survival (*p* = 0.033) (Fig. 5F). The expression level of PTPRC in tumor tissues of female LUAD patients was lower than that in normal tissues (*p* < 0.05) (Fig. 5G and H), and female LUAD patients with high level of PTPRC exhibited a better overall survival (*p* = 0.006) (Fig. 5I). The expression level of BTK in tumor tissues of male LUAD patients was lower than that in normal tissues (*p* < 0.05) (Fig. 5J and K), and male LUAD patients with high level of BTK exhibited a better overall survival (*p* = 0.035) (Fig. 5L). The expression level of CCR2 in tumor tissues of male LUAD patients was lower than that in normal tissues (*p* = 0.035) (Fig. 5M), yet there was no difference in the expression of CCR2 between paired tumors and adjacent normal tissues (*p* > 0.05) (Fig. 5N). Interestingly, male LUAD patients with a high level of CCR2 exhibited a better overall survival (*p* = 0.011) (Fig. 5O). Associations of these key genes expression with age and TNM stage in LUAD patients of different sexes were showed in Additional file 1: Fig. S1.

### CIBERSORT for estimating TIICs in female and male LUAD

**Fig. 6:**
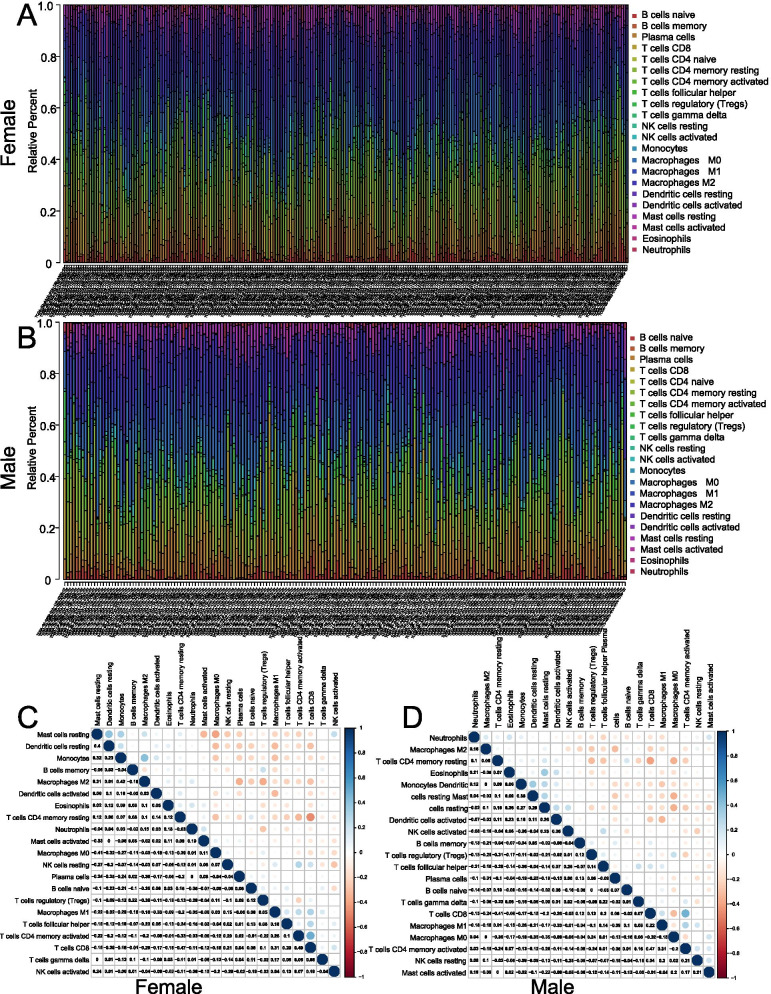
CIBERSORT for estimating TIICs components in TME of female and male LUAD. Stacked bar chart revealing the components of TIICs in female (**A**) and male (**B**) LUAD samples. Correlation matrix indicating the correlation of TIICs in female (**C**) and male (**D**) LUAD samples. A p < 0.05 was considered to be statistically significant

We further selected 22 types of immune cells to explore immune landscape of LUAD. The selected major immune cell types included B cells, T cells, NK cells, neutrophils, dendritic cells, mast cells, macrophages, et al., which participated in regulating process of innate immunity and adaptive immunity. Based on CIBERSORT algorithm, we analyzed the distribution characteristics of the 22 types of immune cells in each LUAD sample (i.e., 270 female patients and 227 male patients) (Fig. 6A and B). In addition, the correlations between various TIICs in female and male LUAD patients varied from weak to moderate (Fig. 6C and D). The correlation matrix indicated that CD8 T cells and M0 macrophages had a strong negative correlation with memory resting CD4 T cells (Cor = −0.47) and resting mast cells (Cor = −0.41) in female LUAD patients. CD8 T cells, M2 macrophages, and resting dendritic cells had a strong positive correlation with memory activated CD4 T cells (Cor = 0.49), monocytes (Cor = 0.42), and resting mast cells (Cor = 0.4) in female LUAD patients (Fig. 6C). As shown in Fig. 6D, for male LUAD patients, there was a significant negative correlation between CD8 T cells and memory resting CD4 T cells (Cor = −0.41), and also a significant positive correlation between CD8 T cells and memory activated CD4 T cells (Cor = 0.47).

### Difference analysis of TIICs in LUAD tumor and adjacent normal tissues

**Fig. 7:**
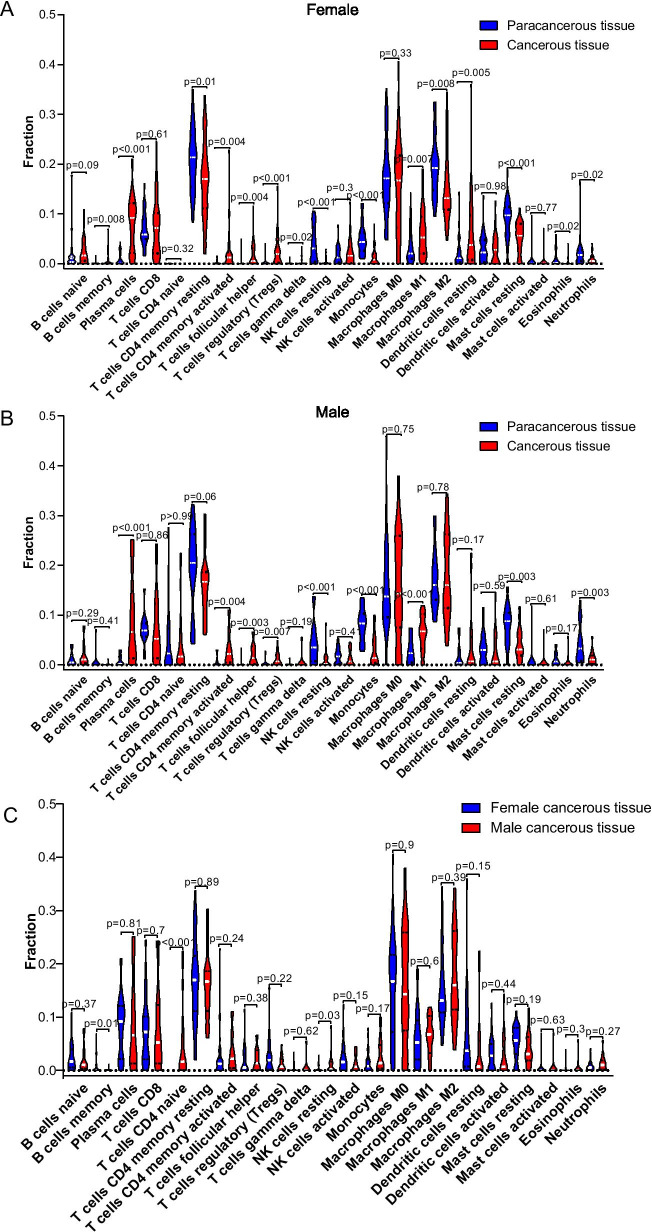
Difference analysis of TIICs in LUAD tumor and adjacent normal tissues. **A** Violin plot showing the proportion of 22 types of TIICs in paracancerous tissues and cancerous tissues in female patients with LUAD. **B** Violin plot showing the proportion of 22 types of TIICs in paracancerous tissues and cancerous tissues in male patients with LUAD. **C** Comparison of the proportion of immune infiltrating cells between female and male patients with LUAD. A p < 0.05 was considered to be statistically significant

To compare the differences in immune cell infiltration between female and male patients with LUAD, we analyzed the characteristics of the distribution of TIICs in TME of female and male patients (Fig. 7). Compared with paracancerous tissues in female patients, 8 types of TIICs (memory B cells, plasma cells, memory activated CD4 T cells, follicular helper T cells, regulatory T cells, gamma delta T cells, M1 macrophages, and resting dendritic cells) had a higher proportion in cancerous tissues, and 6 types of TIICs (memory resting CD4 T cells, resting NK cells, monocytes, M2 macrophages resting mast cells, eosinophils, and neutrophils) had a lower proportion in cancerous tissues (*p* < 0.05) (Fig. 7A). Similarly in male LUAD patients, 5 types of TIICs (plasma cells, memory activated CD4 T cells, follicular helper T cells, regulatory T cells, and M1 macrophages) accounted for a higher proportion in cancerous tissues than those in paracancerous tissues, and the proportion of 4 types of TIICs (resting NK cells, monocytes, resting mast cells, and neutrophils) in cancerous tissues was lower than that in paracancerous tissues (*p* < 0.05) (Fig. 7B). Female patients with LUAD had a higher proportion of memory B cells, while the percentage of T cells CD4 naïve and resting NK cells was lower in female patients (Fig. 7C).

EGFR-mutant LUAD has obvious intratumor heterogeneity [44], which makes it difficult for patients to obtain optimal treatment [45]. We further compared the differences in EGFR-mutation LUAD patients of different sexes, and explored the effect of EGFR mutation on immune cell infiltration. The result indicated that the EGFR mutation rate of female patients was slightly higher than that of male patients (Additional file 1: Fig. S4A). Regardless of female or male patients, there was no significant difference in immune cell infiltration between EGFR-mutant and EGFR-wild patients (Additional file 1: Fig. S4B, C). Interestingly, the proportion of memory B cells in EGFR-mutant female patients was significantly higher than that in EGFR-mutant male patients, while the proportion of Monocytes in the former was lower than that in the latter (Additional file 1: Fig. S4D).

To further research the influence of the proportions of TIICs on prognosis of female and male LUAD patients, univariate Cox regression analysis was conducted on the 22 types of TIICs in female and male patients, respectively. High proportion of activated dendritic cells was identified as risk factor for female LUAD patients (Additional file 1: Table S1). The proportions of gamma delta T cells, activated NK cells and activated mast cells were considered to be related to the prognosis of male LUAD patients (Additional file 1: Table S2).

### Impact of the key identified genes on TIICs

We further analyzed the effects of the main DEGs in female (Additional file 1: Fig. S2) and male (Additional file 1: Fig. S3) patients with LUAD on immune cell infiltration. The relative proportions of memory B cells (*p* = 0.004), CD4 memory resting T cells (*p* = 0.014), resting dendritic cells (*p* = 0.049) and resting mast cells (*p* = 0.011) were significantly upregulated in female patients with high CCR2 expression (Additional file 1: Fig. S2A). However, M0 macrophages (*p* = 0.019) and activated mast cells (*p* = 0.006) were reduced in female patients with high CCR2 expression (Additional file 1: Fig. S2A). The relative proportions of CD 8T cells (*p* = 0.019), CD4 memory activated T cells (*p* = 0.005), M1 macrophages (*p* = 0.009) and neutrophils (*p* = 0.027) were significantly upregulated in female patients with high LCP2 expression (Additional file 1: Fig. S2B). Plasma cells (*p* = 0.002) and M0 macrophages (*p* = 0.038) were decreased in female patients with high LCP2 expression (Additional file 1: Fig. S2B). The relative proportions of memory B cells (*p* = 0.035), CD4 memory activated T cells (*p* = 0.013), and eosinophils (*p* = 0.016) were significantly increased in female patients with high PTPRC expression (Additional file 1: Fig. S2C). Activated NK cells (*p* = 0.044) and activated mast cells (*p* = 0.008) were repressed in female patients with high PTPRC expression (Additional file 1: Fig. S2C).

The relative proportions of memory B cells (*p* = 0.022), CD8 T cells (*p* = 0.034), CD4 memory activated T cells (*p* < 0.001), monocytes (*p* < 0.001), resting dendritic cells (*p* = 0.002), and eosinophils (*p* < 0.001) were significantly upregulated in male patients with high BTK expression (Additional file 1: Fig. S3A). Plasma cells (*p* = 0.024), follicular helper T cells (*p* = 0.031), T cells regulatory (Tregs) (*p* = 0.039), activated NK cells (*p* = 0.013), and M0 macrophages (*p* < 0.001) were inhibited in male patients with high BTK expression (Additional file 1: Fig. S3A). The relative proportions of CD8 T cells (*p* = 0.008), CD4 memory activated T cells (*p* < 0.001), monocytes (*p* = 0.006), and M1 macrophages (*p* < 0.001) were significantly upregulated in male patients with high CCR2 expression (Additional file 1: Fig. S3B). Gamma delta T cells (*p* = 0.031) and M0 macrophages (*p* < 0.001) were repressed in male patients with high CCR2 expression (Additional file 1: Fig. S3B).

### Validation of TCGA results with GEO database

**Fig. 8:**
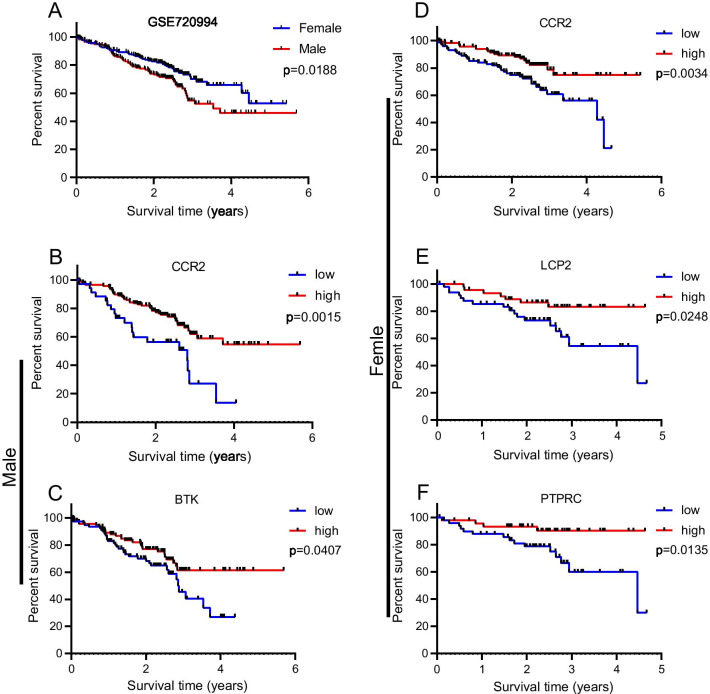
Validation of TCGA results with GEO database. **A** Kaplan–Meier survival curves for female and male patients with LUAD. **B**, **C** Kaplan–Meier plots generated from GEO database to validate the prognosis-related genes (CCR2 and BTK) for male patients in TCGA. **D**–**F** Kaplan–Meier plots generated from GEO database to validate the prognosis-related genes (CCR2, LCP2 and PTPRC) for female patients in TCGA

To verify the prognostic value of the identified genes from TCGA, we used GSE72094 as a validation cohort. Patients were divided into high expression group and low expression group, respectively, according to the specific gene expression. We first compared the overall survival curves of female and male LUAD patients, which showed that the overall survival rate of female patients was better than that of male patients (Fig. 8A). Kaplan–Meier survival curves further confirmed that male LUAD patients with high CCR2 (Fig. 8B) or BTK (Fig. 8C) expression had a significant survival advantage. Similarly, female patients with high CCR2 (Fig. 8D), LCP2 (Fig. 8E), or PTPRC (Fig. 8F) expression had a significant survival advantage.

## Discussion

LUAD is the most common subtype of lung cancer. The incidence and mortality of LUAD in female and male patients are different. The prognosis of male LUAD patients is usually worse than female patients, but the cause is currently unknown. Utilizing TCGA database, this study was the first to compare the differences in the infiltration of immune cells in TME between female and male patients with LUAD, which provided in-depth insights for clarifying the reasons for differences in the prognosis of patients of different sexes.

The ESTIMATE algorithm has been widely used in recent years to evaluate the immune score and stromal score, which can help scholars understand the TME of LUAD in depth. Our results indicated that the estimate score ranged from −2358.46 to 4889.83 among female patients with LUAD and from −2328.69 to 4818.63 among male patients. Similarly, immune score ranged from −943.17 to 3229.35 among female patients with LUAD and from −541.75 to 3441.78 among male patients. Stromal score ranged from −1790.23 to 2097.27 among female patients with LUAD and from −1786.94 to 1722.70 among male patients. In general, average estimate, immune and stromal scores of female patients with LUAD were higher than those of male patients. Interestingly, except that female patients with high estimate score had better overall survival than that with low estimate score, there was no significant difference in overall survival among other high-score and low-score female or male patients. Early research showed that immune-hot tumors were defined as those in which many immune cells such as T cells, lymphocytes et al. had a high proportion, and these infiltrating immune cells improved efficiency of tumor to response to treatment of immune checkpoint inhibitors [46–48]. More immune-hot tumor means higher immune score in the TME. However, cancer patients with higher immune score do not always mean more immune-hot tumor. In this study, female patients presented higher immune score compared with male patients with LUAD. As showed in Fig. 7, compared with male patients with LUAD, female patients had a higher proportion of memory B cells, while the percentage of naïve CD4+ T cells and resting NK cells were lower in female patients, and most immune cells, including naïve B cells, plasma cells, CD8+ T cells, resting/activated memory CD4+ T cells, M0/M1/M2 macrophages, resting/activated dendritic cells, follicular helper T cells, regulatory T cells, gamma delta T cells, activated NK cells, monocytes, resting/activated mast cells, eosinophils, and neutrophils were not significantly different between female and male patients. Hence, although female and male LUAD patients had differences in immune cell infiltration in TME, more study had to be conducted to verify tumor sensitivity to immunotherapy.

To further study the difference of immune-related genes between female and male LUAD patients, we divided female (male) LUAD patients into high- and low-score groups. A total of 304 DEGs were identified in female patients and 368 DEGs were identified in male patients (Fig. 1). GO and KEGG analysis for these DEGs indicated that although there were differences in the signaling pathways enriched in female and male patients, they were all closely related to tumor immunity(Fig. 3). We further used PPI and Cox methods to screen out the most critical prognostic immune-related genes in female and male LUAD patients. CCR2, LCP2, and PTPRC were identified as key prognostic factors of female patients, and BTK and CCR2 were identified as key prognostic factors of male patients (Fig. 4).

Next, we compared the infiltration of immune cells in female and male patients with LUAD. Our results indicated 8 types of TIICs had a higher proportion in cancerous tissues compared with paracancerous tissues in female patients (Fig. 7A), and 5 types of TIICs had a higher proportion in cancerous tissues than that in paracancerous tissues in male patients (Fig. 7B). Recent studies demonstrated that patients with B cells enrichment in TME had a better prognosis and immunotherapy response [49–51]. Memory B cells was the basis for humans to have long-lasting immunity. They responded to reencountered antigens by forming germinal centers (GC) and rapidly producing antibodies [52]. It was reported that high density of tumor-infiltrating memory B cells was closely related to superior survival [53–55]. In our study, the density of memory B cells in TME of female patients with LUAD was significantly higher than that in TME of male patients (Fig. 7C), and the proportion of memory B cells in EGFR-mutant female patients was significantly higher than that in EGFR-mutant male patients, which might explain that female patients had a better prognosis than male patients.

### Perspective and significance

Currently, there is no research using omics data to analyze sex differences in patients with LUAD. Sex factor affects the prognosis of cancer patients. However, its role in shaping the TME is rarely reported, especially its effects on regulating immune cell infiltration in the TME is even less known. To give insight into the sex differences in LUAD, we collected information on 551 tumor samples, and explored the differences in immune cell infiltration in the TME of patients of different sexes based on the gene expression matrix of tumor tissues. In this study, we found that memory B cells were significantly enriched in the tumor tissues of female LUAD patients. Memory B cells play an important role in human anti-tumor immunity and its enrichment often indicates a better prognosis. These findings provide a theoretical basis for explaining that the prognosis of female LUAD patients is better than that of male patients. To better display the immunological characteristics of LUAD patients of different sexes, we separately identified the most important immune-related genes that predicted the prognosis of female patients (CCR2, LCP2, and PTPRC) and male patients (BTK and CCR2). Their powerful predictive value was verified in an independent cohort.

## Conclusions

For the first time, we presented a detailed and comprehensive analysis of the tumor microenvironment immune cell infiltration in female and male patients with LUAD. We found differences in the infiltration of immune cells, the expression of prognostic immune-related genes and related signaling pathways in the tumor microenvironment of female and male patients with LUAD. More importantly, female patients with LUAD had a higher proportion of memory B cells compared with male patients, which provided a reliable theoretical basis for explaining the better prognosis of female LUAD patients than that of male patients.

## Supplementary Information

**Additional file 1: Table S1.** Univariable Cox regression analysis of TIICs with OS for female patients in TCGA. **Table S2.** Univariable Cox regression analysis of TIICs with OS for male patients in TCGA. **Fig. S1.** Associations of CCR2 (A), LCP2 (B) and PTPRC (C) expression with age and TNM stage in female patients with LUAD. Associations of BTK (D) and CCR2 (E) expression with age and TNM stage in male patients with LUAD. **Fig. S2.** The effect of CCR2 (A), LCP2 (B) and PTPRC (C) expression on immune cell infiltration in female patients with LUAD. **Fig. S3.** The effect of BTK (A) and CCR2 (B) expression on immune cell infiltration in male patients with LUAD. **Fig. S4.** Effect of EGFR mutation on immune cell infiltration in TME of patients with LUAD of different sexes. (A) Frequency of EGFR mutation in male and female LUAD patients. Effect of EGFR mutation status on immune cell infiltration in female (B) and male (C) LUAD patients. (D) Comparison of the proportion of immune cell infiltration in female LUAD patients with EGFR mutation and the proportion of immune cell infiltration in male LUAD patients with EGFR mutation.

## Data Availability

The data sets used and/or analysed during the current study are available from the corresponding author on reasonable request.
