# Supplementary material for: Prognostic value of immune-related genes and comparative analysis of immune cell infiltration in lung adenocarcinoma: sex differences
Source: Biol Sex Differ. 2021 Dec 4;12:64. doi: 10.1186/s13293-021-00406-y (PMC8642994; doi:10.1186/s13293-021-00406-y)
Supplement: Supplementary file 1 — Additional file 1: Table S1. Univariable Cox regression analysis of TIICs with OS for female patients in TCGA. Table S2. Univariable Cox regression analysis of TIICs with OS for male patients in TCGA. Fig. S1. Associations of CCR2 (A), LCP2 (B) and PTPRC (C) expression with age and TNM stage in female patients with LUAD. Associations of BTK (D) and CCR2 (E) expression with age and TNM stage in male patients with LUAD. Fig. S2. The effect of CCR2 (A), LCP2 (B) and PTPRC (C) expression on immune cell infiltration in female patients with LUAD. Fig. S3. The effect of BTK (A) and CCR2 (B) expression on immune cell infiltration in male patients with LUAD. Fig. S4. Effect of EGFR mutation on immune cell infiltration in TME of patients with LUAD of different sexes. (A) Frequency of EGFR mutation in male and female LUAD patients. Effect of EGFR mutation status on immune cell infiltration in female (B) and male (C) LUAD patients. (D) Comparison of the proportion of immune cell infiltration in female LUAD patients with EGFR mutation and the proportion of immune cell infiltration in male LUAD patients with EGFR mutation. [file 13293_2021_406_MOESM1_ESM.docx]

**Additional Tables**

| **Table S1. Univariable Cox regression analysis of TIICs with OS for female patients in TCGA** | | | | | |
| --- | --- | --- | --- | --- | --- |
| **Characteristics** | **Hazard.Ratio** | **CI95** | **P.Value** | |  |
| B cells naive | 0.026 | 0-64.325 | 0.36 | |  |
| B cells memory | 55.394 | 0.019-159800.725 | 0.323 | |  |
| Plasma cells | 0.778 | 0.052-11.596 | 0.855 | |  |
| T cells CD8 | 1.125 | 0.04-31.494 | 0.945 | |  |
| T cells CD4 naive | / | / | / | |  |
| T cells CD4 memory resting | 0.128 | 0.008-1.997 | 0.143 | |  |
| T cells CD4 memory activated | 0.774 | 0.002-280.378 | 0.932 | |  |
| T cells follicular helper | 17.935 | 0.006-57379.132 | 0.483 | |  |
| T cells regulatory Tregs | 44.97 | 0.076-26696.993 | 0.243 | |  |
| T cells gamma delta | 0.006 | 0-783365.203 | 0.589 | |  |
| NK cells resting | 0.286 | 0-639492.721 | 0.867 | |  |
| NK cells activated | 0.617 | 0-4462.391 | 0.915 | |  |
| Monocytes | 0.077 | 0-217.283 | 0.528 | |  |
| Macrophages M0 | 3.211 | 0.488-21.147 | 0.225 | |  |
| Macrophages M1 | 4.927 | 0.092-262.843 | 0.432 | |  |
| Macrophages M2 | 1.039 | 0.041-26.113 | 0.981 | |  |
| Dendritic cells resting | 0.116 | 0.004-3.048 | 0.196 | |  |
| Dendritic cells activated | 132.623 | 1.12-15703.463 | 0.045 | |  |
| Mast cells resting | 0.012 | 0-4.473 | 0.142 | |  |
| Mast cells activated | 82816.094 | 0-20364819411663 | 0.251 | |  |
| Eosinophils | 2.335 | 0-1.02717657907191e+23 | 0.975 | |  |
| Neutrophils | 11.23 | 0-2055610429.052 | 0.803 | |  |
|  |  |  | |  | |

| **Table S2. Univariable Cox regression analysis of TIICs with OS for male patients in TCGA** | | | |
| --- | --- | --- | --- |
| **Characteristics** | **Hazard.Ratio** | **CI95** | **P.Value** |
| B cells naive | 0.173 | 0-1253.651 | 0.699 |
| B cells memory | 0 | 0-56.98 | 0.12 |
| Plasma cells | 0.446 | 0.028-7.204 | 0.57 |
| T cells CD8 | 0.107 | 0.004-3.014 | 0.189 |
| T cells CD4 naive | / | / | / |
| T cells CD4 memory resting | 1.733 | 0.095-31.775 | 0.711 |
| T cells CD4 memory activated | 34.451 | 0.013-88179.443 | 0.377 |
| T cells follicular helper | 0.024 | 0-350.486 | 0.446 |
| T cells regulatory Tregs | 0.063 | 0-184.553 | 0.497 |
| T cells gamma delta | 383364.492 | 2.428-60522748381.642 | 0.035 |
| NK cells resting | 128.018 | 0-194411830.42 | 0.504 |
| NK cells activated | 37622.459 | 1.95-725918757.323 | 0.036 |
| Monocytes | 0 | 0-6.909 | 0.111 |
| Macrophages M0 | 1.588 | 0.262-9.623 | 0.615 |
| Macrophages M1 | 33.439 | 0.204-5493.063 | 0.178 |
| Macrophages M2 | 5.406 | 0.256-114.293 | 0.278 |
| Dendritic cells resting | 0.057 | 0.001-3.613 | 0.176 |
| Dendritic cells activated | 3.905 | 0.003-5938.277 | 0.716 |
| Mast cells resting | 0.741 | 0.002-312.963 | 0.923 |
| Mast cells activated | 127892.949 | 1.061-15420616131.403 | 0.049 |
| Eosinophils | 869494331.7 | 0-3.85579514707205e+22 | 0.199 |
| Neutrophils | 0.687 | 0-670294.865 | 0.957 |
|  |  |  |  |

**Additional Figures**


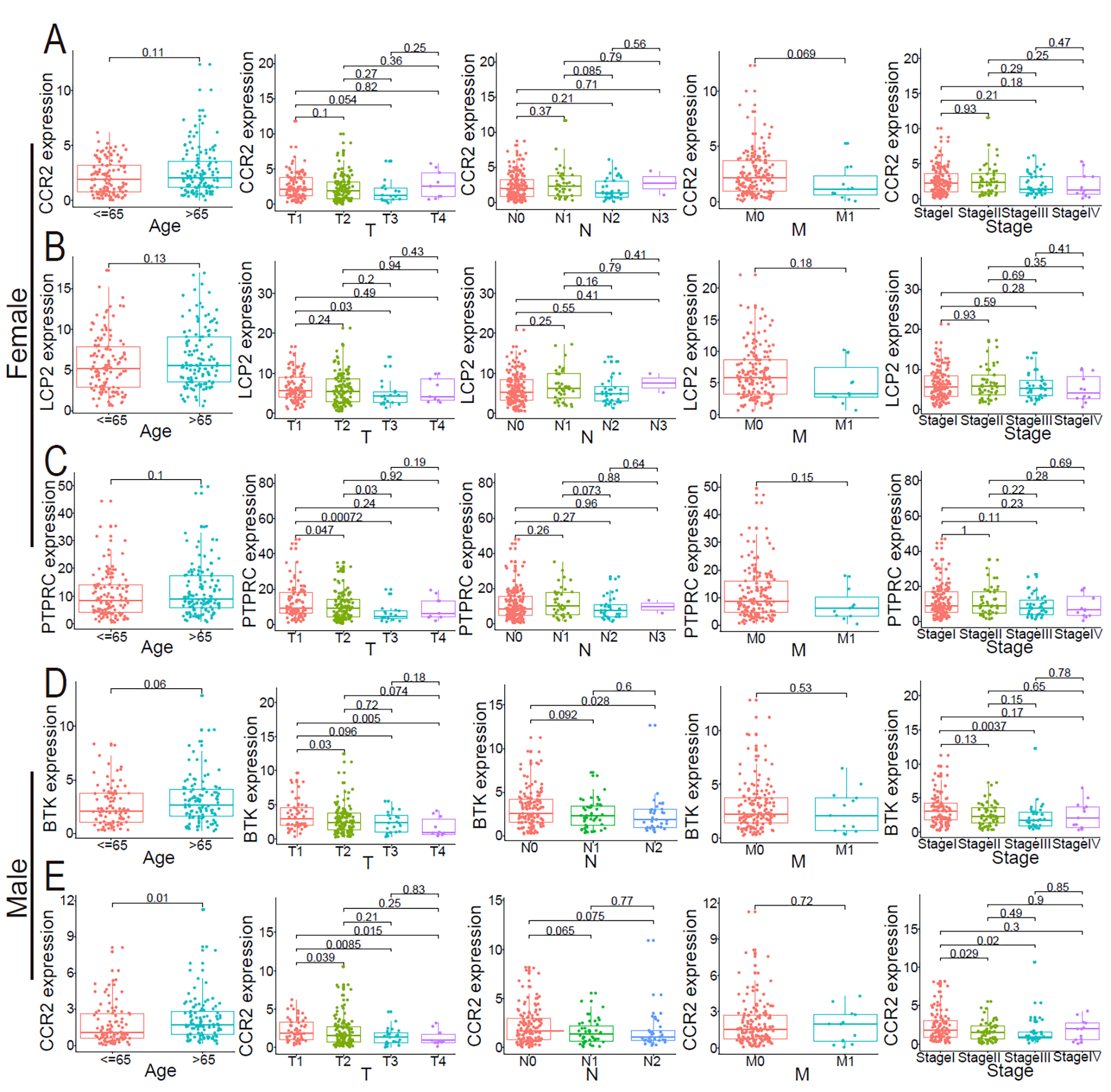


**Figure S1.** Associations of CCR2 (A), LCP2 (B) and PTPRC (C) expression with a**ge** and TNM stage in female patients with LUAD. Associations of BTK (D) and CCR2 (E) expression with a**ge** and TNM stage in male patients with LUAD.


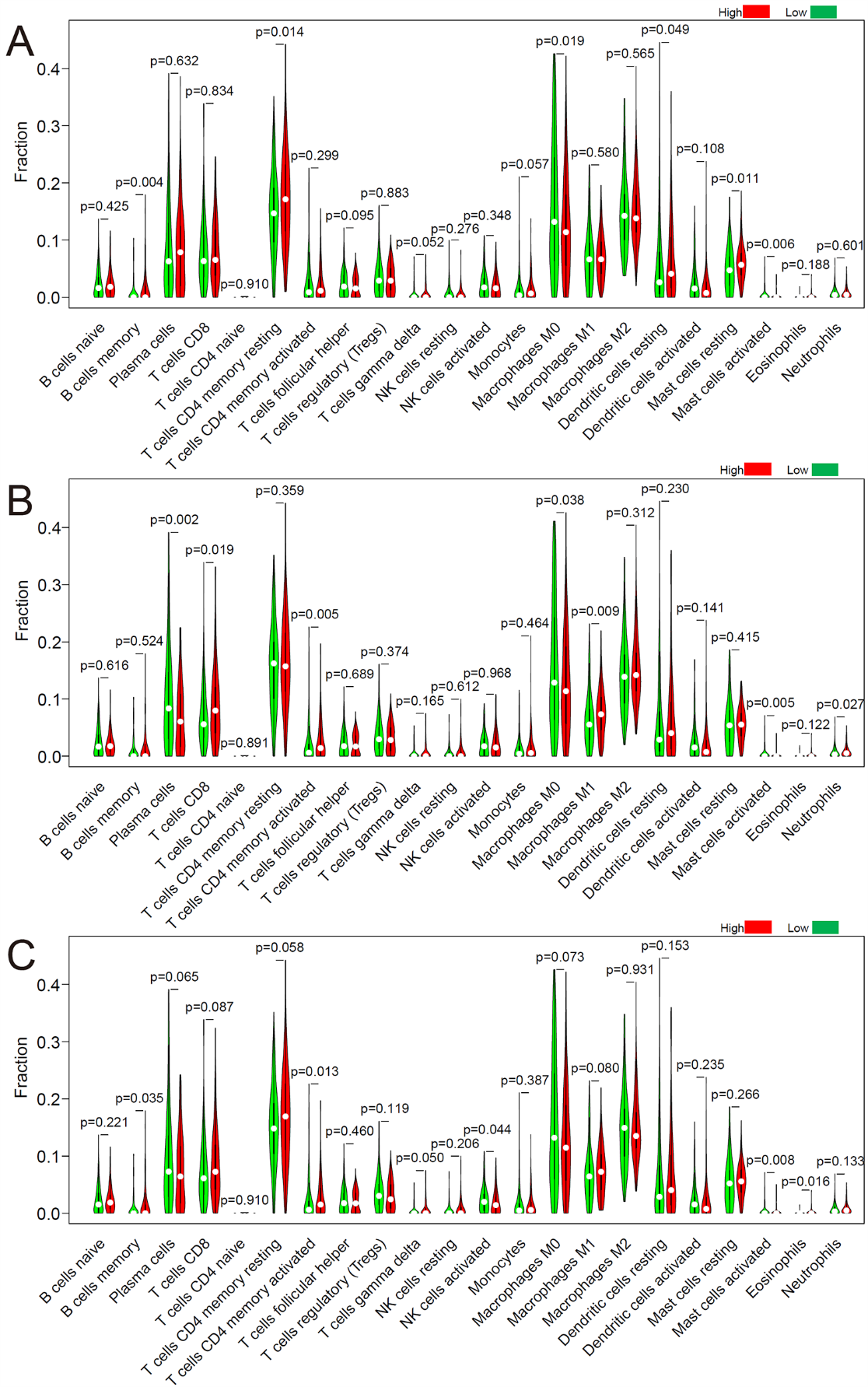


**Figure S2.** The effect of CCR2 (A), LCP2 (B) and PTPRC (C) expression on immune cell infiltration in female patients with LUAD.


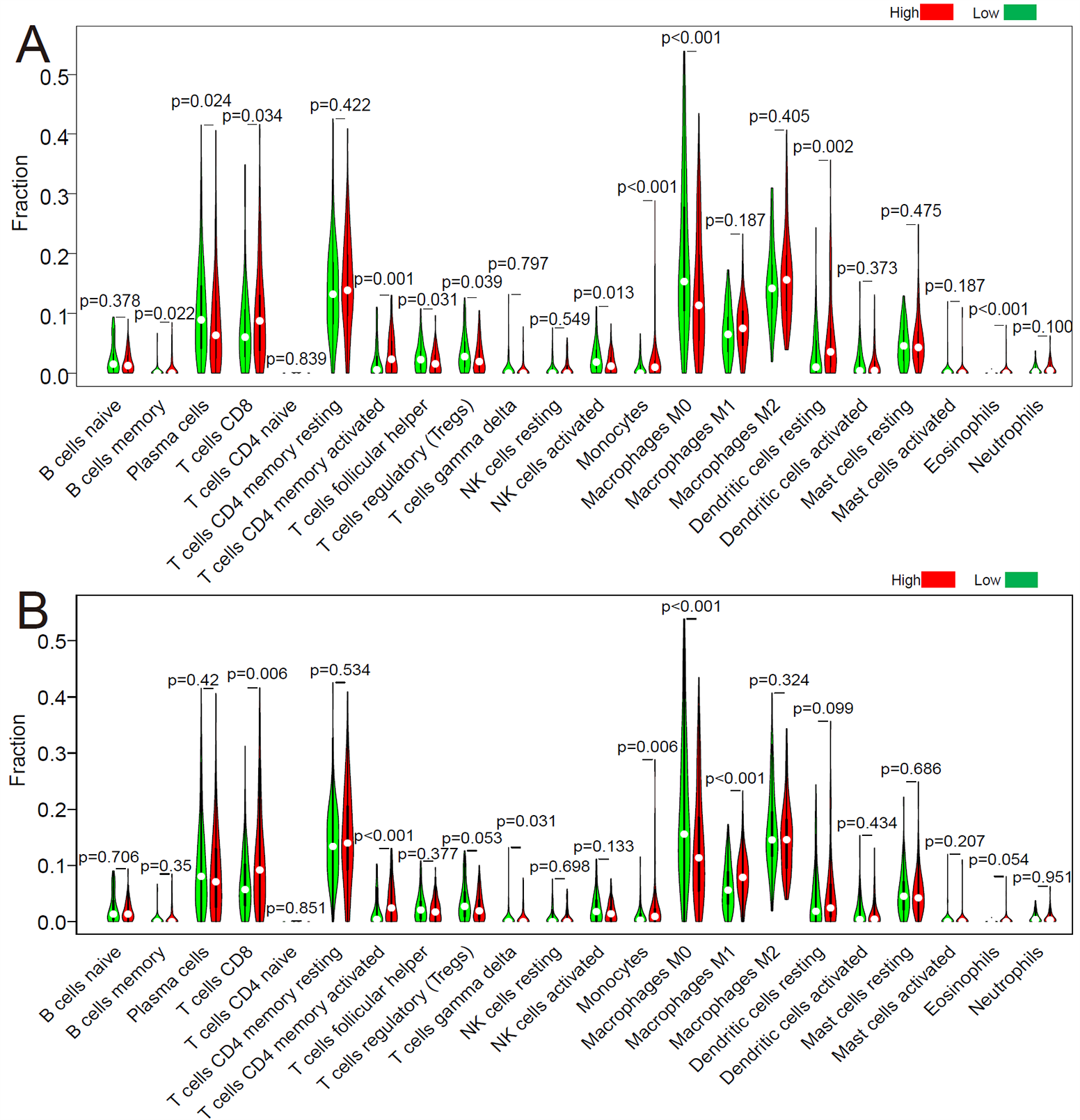


**Figure S3.** The effect of BTK (A) and CCR2 (B) expression on immune cell infiltration in male patients with LUAD.


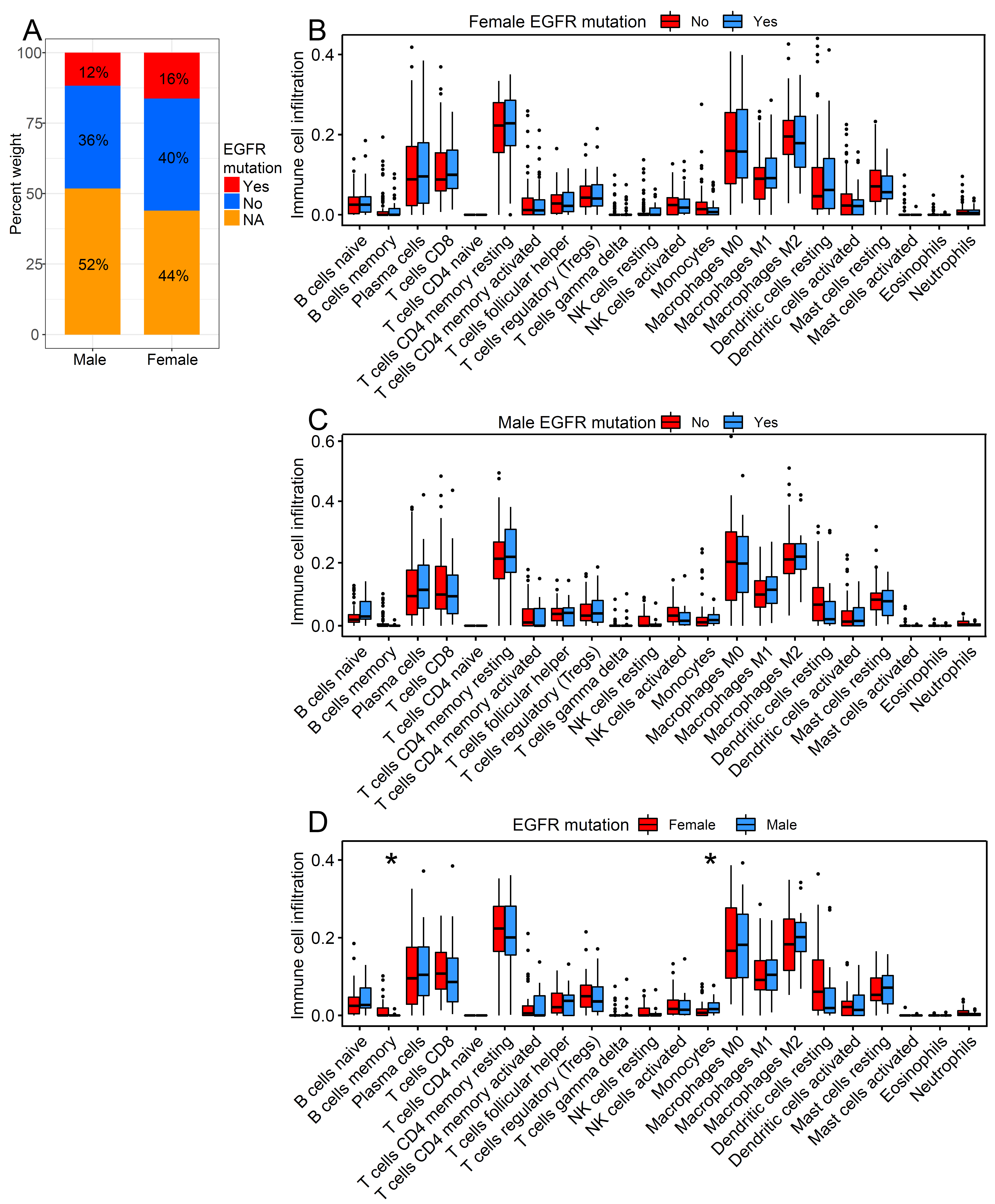


**Figure S4.** Effect of EGFR mutation on immune cell infiltration in TME of patients with LUAD of different sexes. (A) Frequency of EGFR mutation in male and female LUAD patients. Effect of EGFR mutation status on immune cell infiltration in female (B) and male (B) LUAD patients. (D) Comparison of the proportion of immune cell infiltration in female LUAD patients with EGFR mutation and the proportion of immune cell infiltration in male LUAD patients with EGFR mutation.
